# A Meta-Analysis of the Clinical Efficacy of TCM Decoctions Made from Formulas in the Liuwei Dihuang Wan Categorized Formulas in Treating Diabetic Nephropathy Proteinuria

**DOI:** 10.1155/2018/2427301

**Published:** 2018-09-26

**Authors:** Xue Gao, Jianwei Shang, Hongfang Liu, Borui Yu

**Affiliations:** ^1^Dongzhimen Hospital, Beijing University of Chinese Medicine, Beijing 100700, China; ^2^Beijing University of Chinese Medicine, Beijing 100029, China

## Abstract

**Objective:**

Diabetic nephropathy (DN) is one of the microvascular complications of diabetes mellitus. Proteinuria is the most important clinical characteristics of DN and an independent risk factor for disease progression of DN; reducing urine protein is the primary target of treatment strategies for DN. Liuwei Dihuang Wan Categorized Formulas (LDWCFs), a group of classic traditional Chinese medicine (TCM) formulas aiming at “kidney -tonifying”, have been widely used in the treatment of DN. This study aimed to obtain a comprehensive understanding of the TCM method “kidney-tonifying” in the treatment of DN by conducting a meta-analysis to analyze the clinical efficacy of decoctions made from the LDWCFs in the treatment of DN proteinuria.

**Methods:**

CNKI, Wanfang, Weipu, CBM, PubMed, Embase, and the Cochrane Library were searched; 14 studies were included in the meta-analysis.

**Results:**

The results showed that the overall efficacy of the LDWCFs in treating DN was significantly better than that of the comparators (OR 2.87, 95% CI 1.98–4.15,* P*<0.00001). These formulas showed better efficacy than the comparators in reducing 24-hour urinary protein level (MD 0.12, 95% CI 0.06–0.17,* P*<0.0001) and in reducing urine microalbumin excretion rate (SMD 0.87, 95% CI 0.41–1.32,* P*<0.0002). No serious adverse reactions were reported.

**Conclusions:**

TCM formulas included in the LDWCFs are safe and effective in the treatment of DN proteinuria. These findings suggested that the TCM therapeutic principle of “kidney-tonifying” is a valuable addition to the treatment strategies for DN.

## 1. Introduction

Diabetic nephropathy (DN) is one of the most common and serious chronic complications of diabetes mellitus (DM) [1]. Proteinuria is the most important clinical characteristic of DN and an independent risk factor of disease progression [2]; the presence of microalbuminuria can increase all-cause mortality in patients with DM [3]. Current therapeutic strategies for DN are based on lifestyle and diet habits changing, blood glucose and blood pressure control, and dyslipidemia correction [4]. Although many studies in recent years have confirmed the therapeutic effect of various medications including vitamin D [5] and immunosuppressant tacrolimus [6, 7] on reducing DN proteinuria and delaying disease progression, only the angiotensin-converting-enzyme inhibitors (ACEI)/angiotensin receptor blockers (ARB) are recommended for clinical use. However, the use of ACEI/ARBs has limitations. It has been found that ACEI/ARBs are beneficial for patients with diabetics and hypertension who have an estimated glomerular filtration rate (eGFR) of <60 mL/min/1.73 m^2^ and a urinary albumin-creatinine ratio (UACR) of *⩾*300 mg/g. For these patients, ACEI/ARBs can delay the progression of chronic kidney disease (CKD). For patients with normal blood pressure, ACEI/ARBs are not preventive medication for diabetes-induced kidney injury [4].

Traditional Chinese medicine (TCM) demonstrates definite clinical efficacy in the treatment of DN proteinuria. Various TCM extracts or compound preparations have shown confirmed protective effects on the kidney [8–11]. According to the theory of TCM, the disease origin of DN is mainly in the “kidney”; the debilitating “kidney” and the stagnation of the “kidney collateral” are critical to the pathogenesis of DN [12–16]. So the formula prescription principle “kidney-tonifying” plays an important role in the treatment of DN. TCM formulas included in the Liuwei Dihuang Wan Categorized Formulas (LDWCFs) are classical reputable recipes used in TCM. The key ingredients of these formulas are prepared (dried and cooked) root of* Rehmannia glutinosa*, fruit of common* Macrocarpium* (syn.* Cornus*), underground tuber of* Dioscorea batatas* (Chinese yam), dried root bark of* Paeonia suffruticosa* (tree peony), prepared sclerotium of* Wolfiporia extensa* (syn.* Poria cocos*), and dried rhizome of* Alisma plantago-aquatica* (common water-plantain). Zhang Zhongjing (approx. AD 150-219) proposed the use of Shenqi Wan for the treatment of DN in his book “Synopsis of Prescriptions of the Golden Chamber”. By the Song Dynasty, Qian Yi (approx. AD 1032-1117) modified the formula of Shenqi Wan and developed the formula of Liuwei Dihuang Wan. In the following centuries, various formulas such as Zhi Bai Dihuang Wan, Gui Fu Dihuang Wan, and Jisheng Shenqi Wan were derived. These formulas consist of the LDWCFs, aiming on treating the “kidney” with different therapeutic focuses, i.e., “fortifying and replenishing kidney yin”, “fortifying and replenishing kidney yang”, or “fortifying and replenishing kidney qi”.

Since ancient times, LDWCFs have been the most widely used TCM formulas in the treatment of DN [17, 18]. Experimental studies confirmed that LDWCFs has a protective effect on renal interstitial and glomerular injuries [19–23]. Therefore, in order to obtain a comprehensive understanding of the TCM “kidney-tonifying” method in the treatment of DN, a meta-analysis was carried out to analyze the clinical efficacy of decoctions made from the LDWCFs in the treatment of DN proteinuria.

## 2. Materials and Methods

### 2.1. Database Search

In this study, the following databases were searched: CNKI (http://www.cnki.net/), Wanfang (http://www.wanfangdata.com.cn/index.html), Weipu (http://www.cqvip.com/), Chinese Biomedical Literature Database (CBM, http://www.sinomed.ac.cn/), PubMed, Embase, and Cochrane Library. The time frame was from the inception of the individual database to February 2018. The search terms used included (“diabetic nephropathy” OR “diabetic renal disease” OR “diabetic proteinuria”) AND (“dihuangwan” OR “liuweidihuangwan” OR “renqiqiwan” OR “jinkuishenqiwan”) AND “randomized control.”

### 2.2. Inclusion Criteria

Publications meeting the following criteria were included: (1) Articles reported randomized or semirandomized controlled trials. (2) DN diagnostic standards were used and patients were classified as Stage III-IV DN according to the Mogensen DN staging system. (3) The interventions were (a) basic treatment + ACEI/ARB + TCM decoctions from LDWCFs versus basic treatment + ACEI/ARB + TCM placebo (a comparison of the therapy combination and ACEI/ARB alone); (b) basic treatment + TCM decoctions from LDWCFs versus basics Treatment + ACEI/ARB + TCM placebo; or (c) basic treatment + TCM decoctions from LDWCFs versus basic treatment + TCM placebo. Basic treatment included life style change and medications for blood glucose control.

### 2.3. Exclusion Criteria

Publications meeting the following criteria were excluded: (1) The target population did not meet the diagnostic criteria for DN Stages III-IV according to the Mogensen staging system. (2) Interventions included Chinese traditional patent medicine. (3) An excessive number of variables were evaluated. (4) No control group was included, or the control group was not designed to meet the principles of randomization or semirandomization. (5) Blood glucose control in the treatment group was not the same as in the control group at the time of enrollment. (6) Data were not available for analysis or the article was identified as a repetitive publication.

### 2.4. Outcomes

Outcomes measures included 24-hour urinary protein excretion, urinary microalbumin excretion (UAER), fasting plasma glucose (FBG), glycosylated hemoglobin (HbA_1c_), and total effective rate.

### 2.5. Literature Screening and Data Extraction

An EndNote database was built to include all the publications from the searches. Two investigators (Gao X and Yu BR) independently conducted a preliminary screening of the title and the abstract of each article included in the EndNote database. After the preliminary assessment, the full text of the selected publications was evaluated and studies that met the inclusion criteria were further assessed. When the two investigators had different opinions on a certain publication, a third investigator (Liu HF) made an independent assessment and provided the final judgment.

Two investigators (Gao X and Shang JW) independently extracted information from the selected publications. The extracted contents included author (year), sample size, disease stage, patient age, interventions, comparators, treatment period, observation parameters, and decoction composition.

### 2.6. Quality Assessment

Two investigators (Gao X and Shang JW) independently evaluated the quality of the selected publications using the Cochrane Collaboration's risk of bias tool. Assessments included random sequence generation, allocation concealment, blinding, incomplete outcome data, selective outcome reporting, and other possible biases. Using relevant criteria listed in The Cochrane Handbook for Systematic Reviews of Interventions, the publications were categorized as “low risk of bias”, “high risk of bias”, and “unclear risk of bias”.

### 2.7. Statistical Analysis

Meta-analysis was performed using RevMan 5.3. Relative risk (RR) or odds ratio (OR) and a 95% confidence interval (CI) were used for dichotomous variables. Weighted mean difference (WMD) or standardized mean difference (SMD) and a 95% CI were used for continuous variables. The difference was considered statistically significant if *P*<0.05. Chi-square test was used for heterogeneity analysis. If* P⩾*0.1, the difference was considered not statistically significant. When* P*<0.1, if I^2^ > 50%, a random effect model was applied; for any other conditions, a fixed effect model was used.

## 3. Results

### 3.1. Search Results

**Figure 1:**
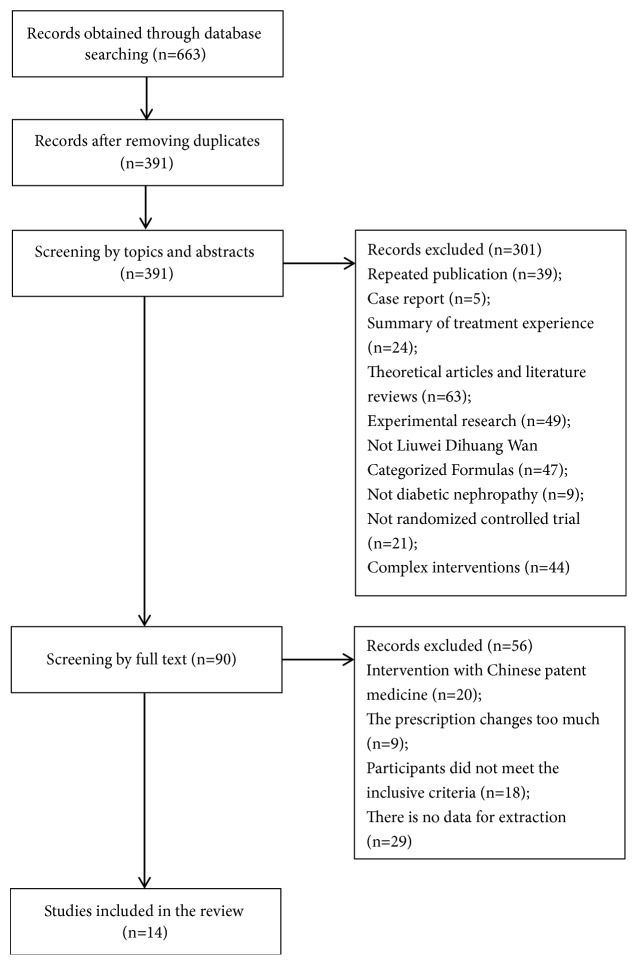
Study selection process.

A total of 663 articles were retrieved, 272 duplicate documents were removed, and 391 articles were included for primary screening. After the primary screening, 301 articles were excluded, and 90 articles were subjected to full-text assessment. Seventy-six articles were excluded after the assessment and 14 articles were included in the meta-analysis.  Figure 1 shows the details.

### 3.2. Basic Characteristics of the Included Studies

All 14 studies were published between 2003 and 2016. A total of 918 study participants were included, 464 in the treatment group and 454 in the control group. All 14 studies included the corresponding basic treatments in the treatment strategy. Various TCM formulas were used in the 14 studies, 8 studies used Liuwei Dihuang Wan, 1 study used Zhibai Dihuang Wan, 2 studies used Jinkui Shenqi Wan, and 3 studies used Jisheng Shenqi Wan. The other TCM formulas included in the LDWCFs were not used. See Table 1 for details.

### 3.3. Study Quality

Among the 14 studies, 3 articles described the method used to generate the random sequence, and 1 article described the distribution concealment method (the decoction was prepared by the hospital's TCM pharmacy and an unidentifiable packaging was used). Blinding was not reported in any of the studies. Two articles failed to report some of the outcome measures in the results section. See Table 2 for details.

### 3.4. Clinical Efficacy

#### 3.4.1. Effective Rate

**Figure 2:**
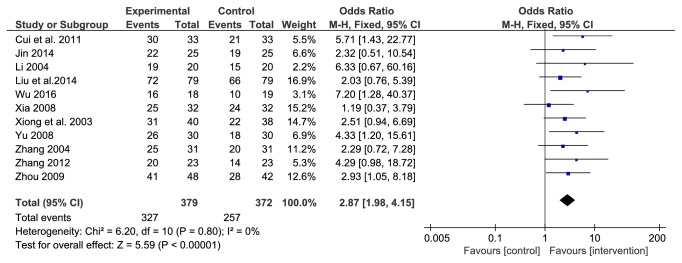
Analysis of total effect rate.

Among the 14 studies, 11 reported the effectiveness of TCM decoctions made from the LDWCFs in the treatment of DN. A total of 751 patients were included, 379 in the treatment group and 372 in the comparator group. Since no heterogeneity was detected (*P*=0.8, I^2^=0%), a fixed effect model was used for the combined analysis. The results showed that the efficacy of the LDWCFs in treating DN was significantly better than that of the comparators (OR 2.87, 95% CI 1.98–4.15,* P*<0.00001) (Figure 2).

#### 3.4.2. Publication Bias of Effective Rate

**Figure 3:**
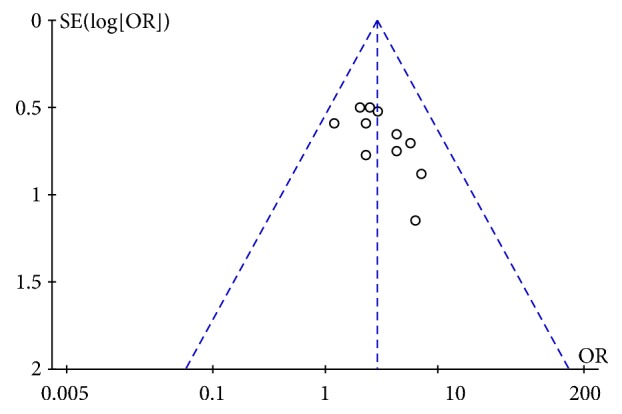
Funnel plot of total effect rate.

We used a funnel plot to show the publication bias of effective rate. The total effective rate of meta-analysis results in intervention and control group was used as abscissa, and the SE(log[OR]) was used as the ordinate. It showed that the funnel plot was not completely symmetrical, which suggested the possibility of publication bias (Figure 3).

#### 3.4.3. 24-Hour Urine Protein Quantitation

**Figure 4:**
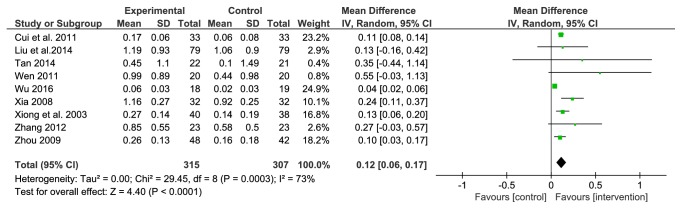
Analysis of 24 h UTP.

24-hour urinary protein quantification was reported in 9 studies. A total of 622 patients were included, 315 in the treatment group and 307 in the comparator group. Due to a substantial heterogeneity among the studies (*P*=0.0003, I^2^=73%), a random effects model was used. The results showed that the efficacy of the LDWCFs was significantly better than that of the comparators (MD 0.12, 95% CI 0.06–0.17,* P*<0.0001) (Figure 4).

In order to analyze the source of heterogeneity, sensitivity analysis was carried out. One of the 9 studies was excluded from the analysis each time, and the results showed that the heterogeneity reduced (*P*=0.38, I^2^=7%) after the removal of the study by Wu JY. Therefore, the study by Wu JY was identified as the source of heterogeneity of this outcome measure (i.e., 24-hour urinary protein quantification). However, when Wu's study was excluded from the meta-analysis, there was no change in the conclusion of the meta-analysis (MD 0.12, 95% CI 0.09–0.15,* P*<0.00001), which indicated that the results are stable and reliable.

#### 3.4.4. Urine Microalbumin Excretion Rate (UAER)

**Figure 5:**
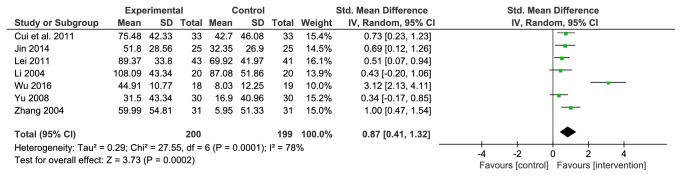
Analysis of UAER.

Seven studies reported UAER, a total of 399 patients were included, 200 in the treatment group and 199 in the comparator group. Since different units (*μ*g/min or mg/24 h) were used in different studies, the SMD was calculated. A random effects model was used as there was substantial heterogeneity among the studies (*P*=0.0001, I^2^=78%). Compared with the comparators, the efficacy of the LDWCFs was significantly better (SMD 0.87, 95% CI 0.41–1.32,* P*<0.0002) (Figure 5).

For the sensitivity analysis, each of the 7 articles was excluded from the analysis and the results showed that there was no substantial heterogeneity among the remaining studies after the removal of the study by Wu JY (*P*=0.55, I^2^=0%). Therefore, Wu JY's study caused the heterogeneity of this outcome measure (i.e., UAER). However, after removing this study, the meta-analysis result did not change (MD 0.61, 95% CI 0.40–0.83,* P*<0.00001), which indicated that the result are stable and reliable.

#### 3.4.5. Fasting Blood Glucose (FBG)

**Figure 6:**
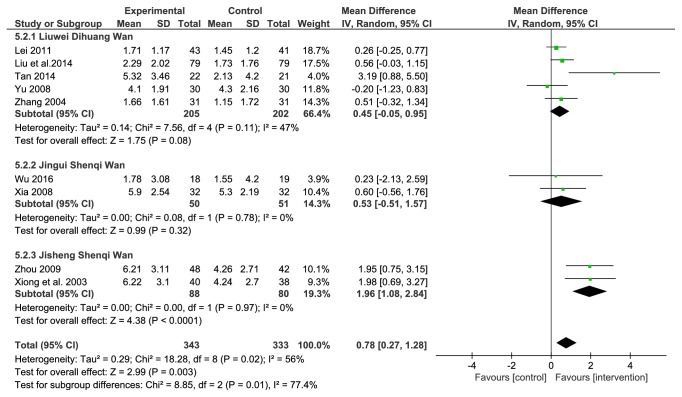
Analysis of FGB.

Nine studies reported fasting blood glucose in the results. A total of 676 patients were included, 343 in the treatment group and 333 in the comparator group. The heterogeneity among the studies was substantial (*P*=0.0002, I^2^=56%), so a random effects model was used. The results showed that the efficacy of the LDWCFs was significantly better than that of the comparators (MD 0.78, 95% CI 0.27–1.28,* P*=0.003) (Figure 6).

In order to analyze the source of heterogeneity, subsequent subgroup analysis was performed. Based on the TCM formula used, the 9 studies were divided into 3 subgroups, the Liuwei Dihuang Wan group (5 studies), the Jinkui Shenqi Wan group (2 studies), and the Jisheng Shenqi Wan group (2 studies). The results showed moderate heterogeneity (*P*=0.11, I^2^=47%) in the Liuwei Dihuang Wan group, while neither the Jinkui Shenqi Wan group (*P*=0.78, I^2^=0%) nor the Jisheng Shenqi Wan group (*P*=0.97, I^2^=0%) was heterogeneous. The combined results showed comparable efficacy of the Liuwei Dihuang Wan group (MD 0.45, 95% CI −0.05 to 0.95,* P*=0.08) and the Jinkui Shenqi Wan group (MD 0.53, 95% CI −0.51 to 1.57,* P*=0.78) versus the comparator group. However, the efficacy of the Jisheng Shenqi Wan group was significantly better than that of the comparator group (MD 1.96, 95% CI 1.08–2.84,* P*<0.0001). (Figure 6). The above results show that the difference in drug components between prescriptions is an important reason for the heterogeneity.

#### 3.4.6. Glycosylated Hemoglobin (HbA1_c_)

**Figure 7:**
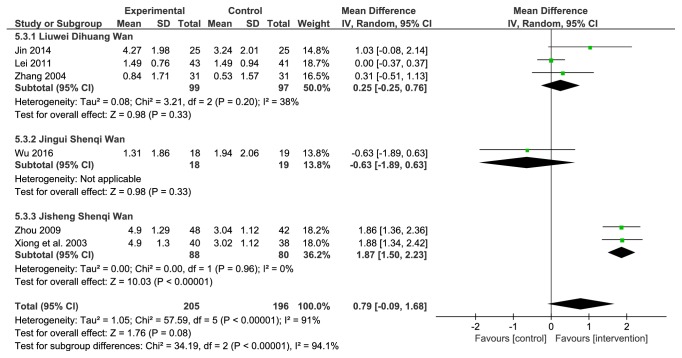
Analysis of HbAlc.

There were 6 studies that included HbA_1c_ in the results. A total of 401 patients were included, 205 in the treatment group and 196 in the comparator group. Because of the substantial heterogeneity among the studies (*P*<0.00001, I^2^=91%), a random effects model was used. The results showed that the efficacy of the LDWCFs was not significantly different from that of the comparators (MD 0.79, 95% CI −0.09 to 1.68,* P*=0.08) (Figure 7).

A subgroup analysis was performed subsequently. The 6 studies were divided into 3 subgroups based on the TCM formula used, including the Liuwei Dihuang Wan group (3 studies), the Jinkui Shenqi Wan group (1 study), and the Jisheng Shenqi Wan group (2 studies). Moderate heterogeneity (*P*=0.20, I^2^=38%) was found in the Liuwei Dihuang Wan group, while the Jisheng Shenqi Wan group was not heterogeneous (*P*=0.96, I^2^=0%). The combined results showed that the efficacy of the Liuwei Dihuang Wan group (MD 0.25, 95% CI −0.25 to 0.76,* P*=0.33) and the Jinkui Shenqi Wan group (MD −0.63, 95% CI −1.89 to 0.63,* P*=0.33) was not significantly different when compared with the comparator group. The efficacy of the Jisheng Shenqi Wan group was significantly better than that of the comparator group (MD 1.87, 95% CI 1.50–2.23,* P*<0.0001) (Figure 7), and the result is similar to fasting blood glucose.

### 3.5. Adverse Events

Among the 14 studies, 3 reported the occurrence or the absence of adverse reactions [21, 27, 29]. None of these 3 studies found any adverse reactions in the treatment group or the comparator group. The remaining studies did not report adverse reactions.

## 4. Discussion

### 4.1. The Significance of This Meta-Analysis

The common point of LDWCFs is to use the large dose of prepared* Radix Rehmanniae* (Shu di huang in Chinese) as the monarch medicine, which can drastically supplement the “kidney essence”. And “kidney essence” is the material basis of “kidney qi”, “kidney yin” as well as “kidney yang”. So they were put together as a class of prescriptions to evaluate the clinical effect of “kidney-tonifying” therapy in the treatment of DN comprehensively. And this is the difference compared with the previous meta-analysis [38].

### 4.2. Summaries of Results

14 randomized controlled trials were analyzed in the present study to evaluate the clinical effect of LDWCFs in DN patients. A total of 918 patients were involved, including 464 patients receiving LDWCFs treatment and 454 patients in the control group. The results showed that the clinical efficacy of routine treatment (including ACER/ARB) combined with LDWCFs was significantly better than of the control group (P<0.01). The treatment combined with LDWCFs decreased the 24-hour urinary protein quantitation (*P*<0.01) and UAER (*P*<0.01) levels. In addition, Jisheng Shenqi Wan (one of LDWCFs) also reduced FBG (*P*<0.01) and HbA1_c_ (*P*<0.01) significantly. These results indicate that LDWCFs may be effective in the treatment of DN.

### 4.3. The Effect of Heterogeneity on Results

From the results of the analysis can be seen, except for the effective rate, all other indicators have significant heterogeneity, which may have resulted from different clinical baseline characteristics and intervention protocols among the included studies. In the subsequent sensitivity analysis, we found that, after rejecting Wu's [23] study, the heterogeneity in 24-hour urine protein quantitative and UAER were eliminated. The specific study had the smallest sample size in all 14 studies and an unreported average age of patients, which may lead to the heterogeneity of the two indicators. However, after excluding Wu's study, the result of meta-analysis did not change, indicating that the results of meta-analysis are stable and reliable.

In the current study, meta-analysis was carried out on fasting blood glucose and HbA1_c_ reported in the 14 studies. Because of the substantial heterogeneity among the included studies on these two outcome measures, subgroup analysis was performed according to the different TCM formulas used. The results showed that the Liuwei Dihuang Wan group and the Jinkui Shenqi Wan group had no treatment advantage on reducing fasting blood glucose and HbA1_c_, while the application of Jisheng Shenqi Wan achieved better hypoglycemic effect compared with the comparator group (Figures 6 and 7). The heterogeneity of Jingui Shenqi Wan and Jisheng Shenqi Wan group was eliminated after grouping the different prescriptions, suggesting that the intervention of different LDWCFs prescriptions may be the reason of the heterogeneity of blood glucose related indicators. It also suggested that the curative effect varies on blood glucose according to different LDWCFs prescriptions, but the curative effect on urinary protein is of no difference.

### 4.4. Limitations

It was shown from the quality evaluation results that the quality of the research included in the current meta-analysis was not satisfying, so the results and conclusions in this study should be interpreted with caution. Firstly, only three of the studies included in this article specifically described the method of randomization, and only one article described allocation concealment method. Thus, it is possible to generate bias in the choice of cases and the distribution of interventions. Secondly, all of the studies did not adopt the blind method, which could lead to performance and detection biases. Thirdly, the placebo effects of prescriptions should not be overlooked because no placebo control was designed in the studies included. At last, as these studies on TCM are all done by Chinese researchers, some negative results are likely tending to be concealed. Therefore, more high-quality multiregional, multicenter, larger-sample RCT studies are needed in the future to make a more comprehensive, real, and objective evaluation of the role of TCM in the treatment of disease.

## 5. Conclusions

Using the LDWCFs alone or in combination with ACEI/ARB may be effective and safe on the treatment of DN proteinuria. However, as mentioned above, due to the large heterogeneity and poor quality of the studies included in this meta-analysis, we cannot give a definite conclusion of the beneficial effectiveness. A large number of standardized clinical trials are still needed to verify this speculation. If the positive effect of LDWCFs is confirmed by more high-level clinical trials in the future, maybe it can become a complementary therapy for DN treatment.

## Figures and Tables

**Table 1:** Characteristics and methodological quality of included studies.

**Reference ** **(year)**	**Participants (T/C)**	**stages of Mogensen**	**Average age (years)**	**Intervention (dosage)**	**Control (dosage)**	**Treatment duration (days)**
Zhou (2009) [24]	90 (48/42)	Phase III	T 55.3; C 53.8	Jisheng Shenqi Wan pluse Captopril (25-75mg/d)	Captopril (25-75mg/d)	42
Zhang (2012) [25]	46 (23/23)	Phase II-IV	T 36.5; C 35.3	Jisheng Shenqi Wan pluse Benazepril Hydrochloride (10mg, qd)	Benazepril Hydrochloride (10mg, qd)	40
Yu (2008) [26]	60 (30/30)	Phase III	T 53±17; C 54±16	Liuwei Dihuang Wan pluse Valsartan (80mg/d)	Valsartan (80mg/d)	84
Xia (2008) [27]	64 (32/32)	Phase IV	T 56.70±4.23; C 55.34±3.33	Jingui Shenqi Wan pluse Valsartan (80mg/d)	Valsartan (80mg/d)	30
Xiong et al. (2003) [28]	78 (40/38)	Phase IV	T 46.35±4.17; C 43.58±4.16	Jisheng Shenqi Wan pluse Captopril (25-75mg/d)	Captopril (25-75mg/d)	42
Wu (2016) [29]	37 (18/19)	Phase III	——	Jingui Shenqi Wan	Routine treatment	30
Wen (2011) [30]	40 (20/20)	Phase IV	T 52.5±11.5; C 50.1±10.7	Liuwei Dihuang Wan pluse Astragalus membranaceus pluse Captopril (25mg/d)	Captopril (25mg/d)	28
Tan (2014) [31]	43 (22/21)	Phase IV	T 59.72±3.17; C 61.32±2.27	Liuwei Dihuang Wan pluse Benazepril Hydrochloride (10mg, qd)	Benazepril Hydrochloride (10mg, qd)	56
Liu et al. (2014) [32]	158 (79/79)	Phase IV	T 57.5±20.1; C 58.6±19.3	Liuwei Dihuang Wan	Routine treatment	60
Li (2004) [33]	40 (20/20)	Phase III	T 51.3; C 52.3	Liuwei Dihuang Wan pluse Enalapril (10mg/d)	Enalapril (10mg/d)	84
Lei (2011) [34]	84 (43/41)	Phase III	T 55.44±5.98; C 55.95±6.99	Liuwei Dihuang Wan	Telmisartan (80mg/d)	84
Jin (2014) [35]	50 (25/25)	Phase III	T 40±13.5; C 38±12.3	Liuwei Dihuang Wan pluse Valsartan (80mg/d)	Valsartan (80mg/d)	90
Cui et al. (2011) [36]	66 (33/33)	Phase III	52.3	Liuwei Dihuang Wan	Routine treatment	56
Zhang (2004) [37]	62 (31/31)	Phase III	T 59.8; C 58.4	Zhibai Dihuang Wan	Routine treatment	90

**Table 2:** Quality assessment of included randomized controlled trials.

**No.**	**Included trials**	**Random sequence generation**	**Allocation concealment**	**Blinding**	**Incomplete outcome data**	**Selective reporting**	**Other sources of bias**
1	Zhou (2009) [24]	Unclear	Unclear	High risk	Low risk	Low risk	Unclear
2	Zhang (2012) [25]	Unclear	Unclear	High risk	Low risk	Low risk	Unclear
3	Yu (2008) [26]	Unclear	Unclear	High risk	Low risk	Low risk	Unclear
4	Xia (2008) [27]	Unclear	Unclear	High risk	Low risk	Low risk	Unclear
5	Xiong et al. (2003) [28]	Unclear	Unclear	High risk	Low risk	Low risk	Unclear
6	Wu (2016) [29]	Low risk, table of random numbers	Low risk, unification of drug packaging	High risk	Low risk	Low risk	Unclear
7	Wen (2011) [30]	Unclear	Unclear	High risk	Low risk	Unclear	Unclear
8	Tan (2014) [31]	Unclear	Unclear	High risk	Low risk	Low risk	Unclear
9	Liu et al. (2014) [32]	Unclear	Unclear	High risk	Low risk	Unclear	Unclear
10	Li (2004) [33]	Low risk, table of random numbers	Unclear	High risk	Low risk	Low risk	Unclear
11	Lei (2011) [34]	Unclear	Unclear	High risk	Low risk	Low risk	Unclear
12	Jin (2014) [35]	Low risk, table of random numbers	Unclear	High risk	Low risk	Low risk	Unclear
13	Cui et al. (2011) [36]	Unclear	Unclear	High risk	Low risk	Low risk	Unclear
14	Zhang (2004) [37]	Unclear	Unclear	High risk	Low risk	Low risk	Unclear
